# Acupoint catgut embedding for insomnia: Protocol for a systematic review and data mining

**DOI:** 10.1097/MD.0000000000019333

**Published:** 2020-02-28

**Authors:** Xiaotong Wang, Yinna Huang, Minting Li, Haixiong Lin, Chunni Lin, Weiqin Yang, Xiaopeng Ye

**Affiliations:** a Medical College of Acu-Moxi and Rehabilitation, Guangzhou University of Chinese Medicine; b Foresea Life Insurance Guangzhou General Hospital; c The First School of Clinical Medicine, Guangzhou University of Chinese Medicine, Guangzhou; d School of Foreign Languages, Xinhua College of Sun Yat-sen University, Dongguan; e The Eighth Affiliated Hospital, Sun Yat-sen University; f Shenzhen Bao’an Traditional Chinese Medicine Hospital Group, Guangzhou University of Chinese Medicine, Shenzhen, People's Republic of China.

**Keywords:** acupoint catgut embedding, data mining, insomnia, protocol, systematic review

## Abstract

**Background:**

Insomnia is a common sleep disorder characterized by chronically disturbed sleep or loss of sleep, and even cognitive dysfunction. Acupoint catgut embedding is widely used to treat sleep disorders. However, there is no systematic review and data mining of the effectiveness and potential acupoints prescription of acupoint catgut embedding for insomnia.

**Methods:**

Randomized controlled trials (RCTs) from the Web of Science, PubMed, Cochrane Library, Springer, Wanfang database, China National Knowledge Infrastructure, VIP Chinese Science and Technology Journals Database, and 2 clinical trial registration center will be included. The search time will be established from each database to December 30, 2019. The outcome measures will be Pittsburgh sleep quality index (PSQI), clinical effective rate, International Unified Sleep Efficiency Value (IUSEV) and adverse events. Data from RCTs that meets the inclusion criteria will be analyzed through RevMan V.5.3 software. Risk of bias and publication bias will be analyzed to identify the quality of the included studies. Besides, Traditional Chinese Medicine inheritance support system (TCMISS) will be used to analyze the potential acupoints prescriptions.

**Results:**

This study will clarify PSQI, IUSEV, clinical effective rate, adverse events, and potential acupoint prescriptions of acupoint catgut embedding for patients with insomnia.

**Conclusion:**

Our study will provide evidence of acupoint catgut embedding for insomnia, which may be beneficial to practitioners in the field of non-pharmacological interventions.

PROSPERO registration number: CRD42019144636.

## 1 Introduction

Insomnia is the most common sleep disorder. The incidence of insomnia in adults is as high as 10% to 15%, and most of the symptoms of insomnia often turn into chronic diseases.^[1]^ Nearly half of patients with severe insomnia have a course of more than 10 years.^[1]^ Insomnia seriously impairs the physical and mental health of patients and affects their quality of life. In addition, insomnia imposes a serious mental and economic burden on individuals and society.^[2]^ Common used hypnotic drugs are benzodiazepines, non-benzodiazepines, antidepressants, antihistamines, which have a certain effect on patients with mild or newly diagnosed insomnia.^[1]^ However, these drugs have troublesome side effects, and long-term use is prone to tolerance and drug dependence.^[3]^ Therefore, a growing number of people are seeking complementary and alternative therapy to improve the efficacy of hypnotic drugs and reduce adverse reactions.^[4]^

At present, acupoint catgut embedding as an adjuvant therapy is currently being widely used in different medical centers.^[5]^ And many clinical trial institutions are actively conducting randomized controlled trials (RCTs) to further verify the therapeutic effect and possible mechanism of acupoint catgut embedding on insomnia.^[6]^ However, due to differences in regions, races, and operators, the results of acupoint catgut embedding for insomnia may be different. Therefore, systematic evaluation of treatment outcomes and treatment prescriptions may help to better explain and apply this technology. In this study, we aimed to explore RCTs of acupoint catgut embedding for insomnia to provide efficacy assessments and potential prescriptions for clinical applications.

## 2 Methods

### 2.1 Study type

We will collect RCTs that assessed the PSQI, clinical effective rate, IUSEV or adverse events of acupoint catgut embedding on insomnia. Articles of the following research types will be excluded: case series, qualitative studies, case-control studies, observational studies, animal experiments, review articles. There are no restrictions on study area, race, patient age, and gender.

### 2.2 Participants

Participants with insomnia will be considered in this study. The diagnosis of insomnia needs to be consistent with ICSD-3 in the International Classification of Sleep Disorders II^[7]^ or Guidelines for the diagnosis and treatment of insomnia in China.^[1]^

### 2.3 Interventions

In the intervention group, patients received acupoint catgut embedding. In the control group, patients received medication, no treatment, sham or placebo acupoint catgut embedding, and etc. The other interventions between the control group and the intervention group are the same.

### 2.4 Clinical outcome measures

The primary outcomes will include PSQI and clinical effective rate.

The secondary outcomes will be IUSEV and adverse events.

### 2.5 Search strategy

We will identify relevant clinical trials from the following 7 electronic databases: Web of Science (http://apps.webofknowledge.com/), PubMed (https://www.ncbi.nlm.nih.gov/pubmed/), Cochrane Library (https://www.cochranelibrary.com/), Springer (https://link.springer.com/), Wanfang database (http://www.wanfangdata.com.cn/index.html), China National Knowledge Infrastructure (http://kns.cnki.net), VIP Chinese Science and Technology Journals Database (http://qikan.cqvip.com/). The search time will be established from each database to December 30, 2019. The search terms will include intervention methods, diseases and research types: (‘acupoint catgut embedding’ or ‘acupoint catgut embedding therapy’ or ‘acupoint embedding’ or ‘acupoint therapy’ or ‘thread-embedding acupoint ligation’ or ‘acupoint ligat thread embed’) and (‘insomnia’ or ‘insomnia disorder’ or ‘chronic insomnia’ or ‘short-term insomnia’ or ‘other types of insomnia’ or ‘sleep disorder’) and (‘clinical trial’ or ‘randomized controlled trial’ or ‘randomized’ or ‘trial’) and (‘blind’). Detailed search strategy for Web of Science is showed in Table 1. Similar but slightly adjusted search strategies will be performed in the other 6 databases. The language of publication will be limited to Chinese and English. But it will not limit the race of the participants. To avoid missing ongoing clinical trials, we will search the following two trial registration centers to identify relevant studies: China Clinical Trial Registry: www.chictr.org.cn/index.aspx, Clinical trial: www.ClinicalTrials.gov. Besides, we will screen the references of included studies to identify other potential clinical trials.

**Table 1:**
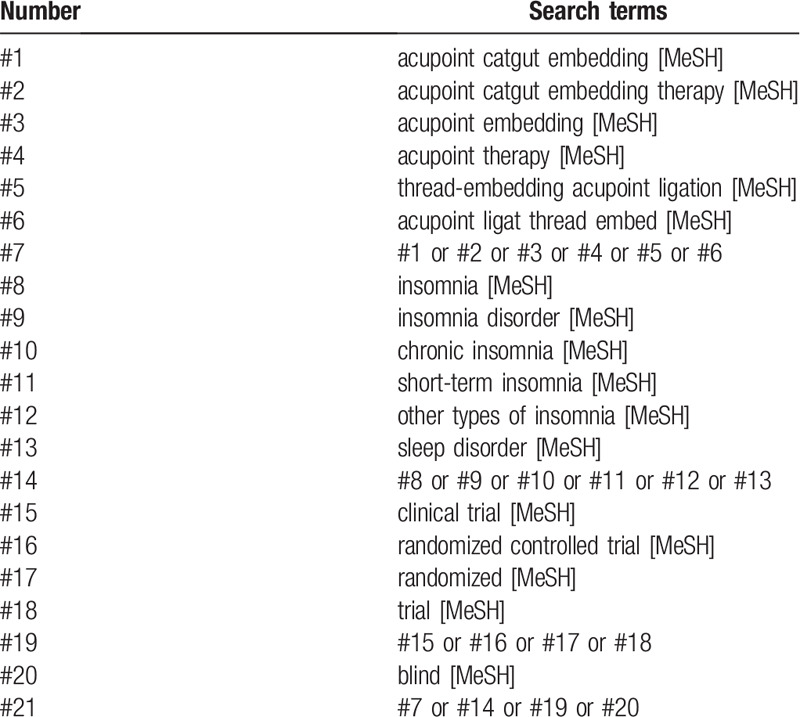
Represents the search strategy for Web of Science.

### 2.6 Study selection and data extraction

**Figure 1:**
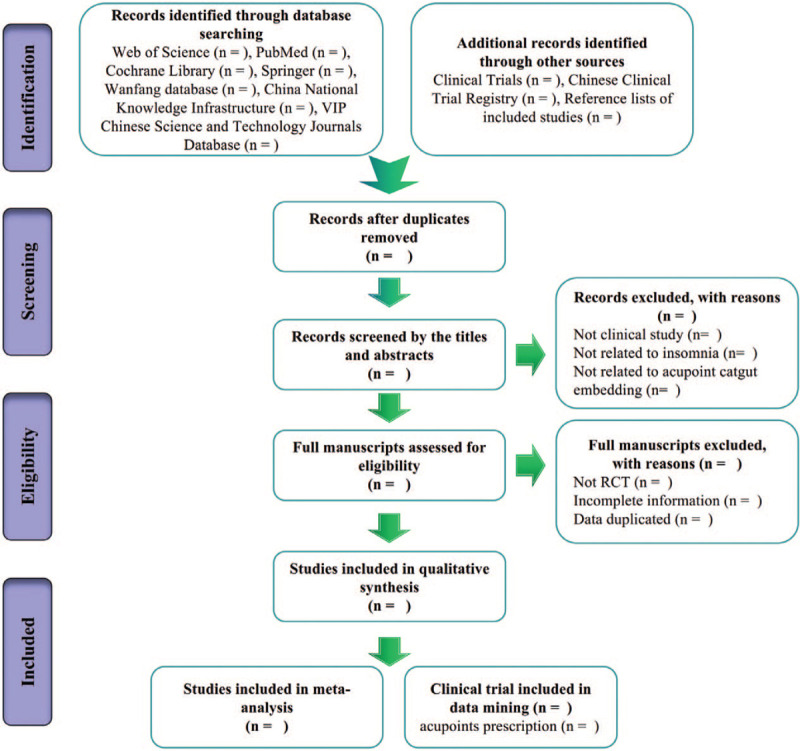
Flow chart of the search process.

Study selection and data extraction will be performed by two authors (Huang YN and Yang WQ). Literature will be managed by the NoteExpress 3.2.0 software. First, use the NoteExpress 3.2.0 software to exclude duplicate references from different databases. Then, two reviewers (Huang YN and Yang WQ) will evaluate the titles and abstracts of each clinical trial to identify the eligible studies independently. Finally, read the full text to assess eligible studies. If there is a disagreement, it will be resolved through discussions between authors. Study selections will be summarized in a Preferred reporting items for systematic review and meta-analysis protocols (PRISMA) flow chart (http://www.prisma-statement.org) (Fig. 1). We will use a pre-designed data extraction table for data extraction. Extracting data includes: the first author, publication year, country where the trial was conducted, number of participants, age of participants, gender, treatment duration, dropout number, outcome, follow-up periods and adverse events.

### 2.7 Dealing with missing data

We will use intention to treat basis to analyze the data, and we will obtain the missing data by contacting the original authors. If detailed information is not available in this way, we will only analyze the available data.

### 2.8 Assessment of risk of bias

Two authors (Lin HX and Wang XT) will assess the study designs and methods using risk of bias recommended by Cochrane Handbook independently. If there is a disagreement, it will be resolved through discussions. The risk of bias includes 6 aspects: random sequence generation, reviewer blind setting, allocation concealment, incomplete outcome data, selective report, and other bias. The risk of bias will be divided into unclear, low and high.

### 2.9 Data synthesis and analysis

We will use the RevMan software (version 5.3) to analyze the data.^[8]^ Dichotomous data and continuous data will be pooled and expressed as risk ratio and mean differences or standardized mean difference with 95% confidence intervals independently. The actual differences reflecting the effect size will be analyzed by *Q* test and *I*^2^ statistic, where the *I*^2^ values represent severe, moderate, and mild heterogeneity (>75%, 25–75%, and <25%, respectively).^[9]^ If there is mild or moderate heterogeneity between studies, the data will be pooled using a fixed-effects model,^[10]^ otherwise a random effects model is used.^[11]^

### 2.10 Additional analyses

If the data is insufficient, we will only perform qualitative synthesis. Meta-regression will be performed to assess the potential heterogeneity. Subgroup analysis will be adopted to investigate the impact of the type of intervention and duration of treatment on the outcome. Sensitivity analysis will be conducted to check the robustness of pooled outcome. TCMISS (Version 2.5) will be used to explore the potential acupoints prescription of acupoint catgut embedding for insomnia.

### 2.11 Assessment of reporting biases

We will assess publication bias by visual inspection of Funnel plots supplemented by Begg and Egger tests, where appropriate.

### 2.12 Confidence in cumulative evidence

The Grading of Recommendations Assessment, Development and Evaluation will be used to assess the quality of design, directness, consistency, precision, and publication bias, which will facilitate the development of the guideline.^[12]^ Each item will be divided into four levels: very low, low, medium, and high.

## 3 Discussion

Insomnia is characterized by chronically disturbed sleep or loss of sleep, and even cognitive dysfunction.^[13]^ Despite its negative impact, less than 15% of patients with chronic insomnia receive treatment.^[14]^ When treatment is initiated, hypnotic drugs became the first choice for insomnia due to the short-term efficacy.^[15]^ However, due to the risk of residual daytime effects and insufficient long-term efficacy of hypnotic drugs, non-drug intervention studies, such as acupoint catgut embedding, are particularly important.^[5,16]^ At present, many clinical trials of acupoint catgut embedding as a separate regimen or combined hypnotic therapy for insomnia are being carried out.^[4,5]^ Previous studies have found that acupoint catgut embedding could up-regulate hypothalamic monoamine neurotransmitter serotonin, IL-1β and TNF-α levels, and down-regulate dopamine and norepinephrine, which may be the mechanism to improve insomnia.^[17]^ To the best of our knowledge, even though acupoint catgut embedding is often used for insomnia, there is no systematic review and data mining of the effectiveness and potential acupoints prescription of acupoint catgut embedding for insomnia. Therefore, we take acupoint catgut embedding as a research object and explore its efficacy, safety and prescription for treating insomnia.

PSQI is a widely used self-reported sleep questionnaire measurement method that could be used to identify the possible causes and nature of insomnia and to provide subscale scores for sleep problem, including sleep duration, latency, efficiency, disturbances, quality, and daytime dysfunction.^[18]^ IUSEV reflects the proportion of actual sleep time in bed time, and is currently widely used to evaluate the effect of acupoint catgut embedding for insomnia.^[19]^ Therefore, we selected PSQI and IUSEV to evaluate the effect of acupoint catgut embedding on insomnia in different studies. Clinical trials of acupoint catgut embedding often use Chinese medicine syndrome scores to assess clinical effective rate of insomnia patients,^[20]^ so we will also consider this indicator. Besides, any medical technology or operation needs to use adverse reactions as an indicator to identify health hazards and avoid irreversible serious incidents.^[21]^ It is reported that common adverse events in acupoint catgut embedding are subcutaneous induration, hematoma, pain, suppuration, allergies, fever, numbness, and even fainting.^[22]^ However, the occurrence of these adverse events in patients with insomnia is not clear, which requires further systematic summary. This study will assess the effect of acupoint catgut embedding on PSQI, IUSEV, clinical effective rate and adverse events in patients with insomnia.

TCMISS, invented by the *Institute of Chinese Materia Medica China Academy of Chinese Medical Sciences,* is widely used to explore potential acupoint prescriptions for different diseases.^[23]^ It is an innovative data mining software for text mining, complex system entropy clustering and association rule analysis, enabling researchers with no algorithmic experience to easily perform big data analysis.^[24]^ Therefore, we will use TCISS to explore acupoint prescriptions for insomnia, which may be beneficial to practitioners in the field of non-pharmacological interventions.

### 3.1 Ethics and dissemination

Since this work is based on published data, no ethical approval is required. The results will be submitted to a peer-reviewed journal.

## 4 Conclusions

Our study will explore PSQI, IUSEV, clinical effective rate, adverse events and potential acupoint prescriptions of acupoint catgut embedding for insomnia, which may be beneficial to practitioners in the field of non-pharmacological interventions.

## Author contributions

**Conceptualization:** Xiaotong Wang, Haixiong Lin.

**Data curation:** Xiaotong Wang, Haixiong Lin, Weiqin Yang.

**Formal analysis:** Xiaotong Wang, Yinna Huang, Minting Li, Haixiong Lin, Chunni Lin, Weiqin Yang.

**Funding acquisition:** Haixiong Lin.

**Investigation:** Xiaotong Wang, Weiqin Yang.

**Methodology:** Xiaotong Wang, Yinna Huang, Chunni Lin, Xiaopeng Ye.

**Resources:** Xiaotong Wang, Yinna Huang, Chunni Lin.

**Software:** Xiaotong Wang, Minting Li.

**Supervision:** Xiaopeng Ye.

**Writing – original draft:** Xiaotong Wang, Haixiong Lin.

**Writing – review & editing:** Minting Li, Xiaopeng Ye.

Xiaotong Wang orcid: 0000-0002-5329-2663.

Haixiong Lin orcid: 0000-0002-9939-7698.
